# Description of *Scheffersomyces henanensis* sp. nov., a New D-Xylose-Fermenting Yeast Species Isolated from Rotten Wood

**DOI:** 10.1371/journal.pone.0092315

**Published:** 2014-03-19

**Authors:** Yongcheng Ren, Liang Chen, Qiuhong Niu, Fengli Hui

**Affiliations:** School of Life Science and Technology, Nanyang Normal University, Nanyang, PR China; National Center for Biotechnology Information (NCBI), United States of America

## Abstract

Two strains of a D-xylose-fermenting yeast species were isolated from rotten wood samples collected from the Baotianman Nature Reserve in Henan Province, central China. These strains formed hat-shaped ascospores in conjugated and deliquescent asci. Multilocus phylogenetic analysis that included the nearly complete small subunit (SSU), the internal transcribed spacer (ITS) region and the D1/D2 domain of the large subunit (LSU) rRNA genes, as well as RNA polymerase II largest subunit (*RPB1*) gene demonstrated that the two strains represent a novel yeast species closely related to *Scheffersomyces segobiensis*. A sequence comparison of xylose reductase (*XYL1*) gene, which was recently recommended for rapid identification of cryptic species in the *Scheffersomyces* clade, revealed a significant sequence divergence of 25 nucleotides between the novel strains and their closest relative *S*. *segobiensis*, supporting their classification as a distinct species. Furthermore, these new strains can be clearly distinguished from *S*. *segobiensis* by a number of morphological and physiological characteristics. Therefore, a novel yeast species, *Scheffersomyces henanensis* sp. nov., is proposed to accommodate these strains. The type strain is BY-41^T^ ( =  CICC 1974^T^  =  CBS 12475^T^).

## Introduction

The genus *Scheffersomyces* was proposed by Kurtzman and Suzuki based on phylogenetic analysis from the combined sequences of the D1/D2 domain of the large subunit (LSU) and the nearly complete small subunit (SSU) rRNA genes [1]. At the time of description, the genus contained three species, *Scheffersomyces stipitis*, *S*. *segobiensis* and *S*. *spartiniae*, which were transferred from the genus *Pichia* [1], [2]. The genus *Scheffersomyces* was later expanded by the inclusion of seven related *Candida* species as new combinations, as well as three novel species, *S*. *illinoinensis*, *S*. *quercinus* and *S*. *virginianus*, which were isolated from rotten wood [3]. Thus, 13 species were included in this genus, which clustered in an independent clade based on a multilocus phylogenetic analysis that included the traditional SSU and LSU markers, the orthologous *RPB1*, and the recently proposed ITS barcoding region for fungi [3], [4]. More recently, several new species of the genus *Scheffersomyces* including *S*. *cryptocercus* [5], *S*. *parashehatae* and *S*. *xylosifermentans* [6] have been recovered from wood-ingesting insects.

Yeasts of the genus *Scheffersomyces* have been found to occupy habitats rich in xylose, including decaying wood [3], [7]–[9], wood-feeding insects [3], [5], [6], [10] and their resulting frass [10], [11]. Many of these yeast species, such as *S*. *cryptocercus*, *S*. *illinoinensis*, *S*. *insectosa*, *S*. *lignosus*, *S*. *quercinus*, *S*. *segobiensis*, *S*. *shehatae*, *S*. *stipitis* and *S*. *virginianus*, possess the rare ability to produce ethanol by fermentation of D-xylose, which gives them economic potential for the production of bioethanol from plant waste residues [12]–[14]. *S*. *shehatae* and *S*. *stipitis* are considered the best ethanol producers among these naturally D-xylose-fermenting yeasts [13], [15]. Despite the existence of these microorganisms, obtaining high ethanol yields from pentose sugars on a large scale remain a challenge [16], as microorganisms that robustly convert pentose sugars into ethanol at high yields while withstanding fermentation inhibitors have not yet been identified [17]. Therefore, there is a need for identifying new yeasts capable of efficient xylose fermentation for bioethanol production. Identification of yeast strains that ferment hemicellulosic sugars will lead to improved prospects for lignocellulosic ethanol production [18]. Such strains can be obtained by isolation from the environment, strain mutation and selection in the laboratory [8], [19] or by engineering strains of *Saccharomyces cerevisiae* capable of fermenting D-xylose [20].

During an investigation of the yeast community associated with rotten wood obtained from the Baotianman Nature Reserve of Henan Province, central China, we isolated two D-xylose-fermenting yeasts whose physiology and ascospore morphology typically resembled those of the genus *Scheffersomyces*. Multilocus phylogenetic analysis and nucleotide sequence comparison of the single copy xylose reductase (*XYL1*) gene indicated that these strains represent a novel yeast species closely related to *S*. *segobiensis*. In this paper, we describe this new species as *Scheffersomyces henanensis* sp. nov.

## Materials and Methods

### Yeast Isolation and Culture

One hundred and five yeast strains were isolated from 23 samples of rotten wood collected from the Baotianman National Nature Reserve in Henan Province, central China (33°27′47′′N and 111°48′32′′E). Strain BY-41^T^ was isolated from a sample collected in a mixed deciduous forest in August 2009, whereas the other strain BY-58 was found in a sample from a deciduous *Quercus* forest in June 2010. The field collections were made according to Chinese diversity rules, and all necessary permits were obtained for the described field studies. Isolation of the strains was carried out by the enrichment technique using yeast extract-malt extract (YM) broth (0.3% yeast extract, 0.3% malt extract, 0.5% peptone, 1% glucose; adjusted to pH 4.0–4.5 with 1 M HCl) supplemented with 0.025% sodium propionate and 200 mg/L chloramphenicol [21]. Representative colonies were purified by the conventional streaking technique on YM agar plates. Purified yeast strains were suspended in YM broth supplemented with 10% glycerol and maintained at −80°C.

### Morphological, Physiological and Biochemical Characteristics

The morphological, physiological and biochemical characteristics were examined according to standard methods that are employed in yeast taxonomy [2], [22], [23]. All assimilation tests were performed three times, and the results were read after 5 and 21 days of incubation. For the examination of ascospores, the strains were incubated on YM agar, McClary’s acetate agar, cornmeal agar and 5% malt extract agar [23], either individually or as pairwise mixtures on the sporulation medium. Ubiquinones were extracted and purified by the method of Yamada and Kondo with slight modifications and determined by HPLC as described previously [24], [25].

### Amplification and Sequencing of DNA

Genomic DNA was extracted with a Dr. GenTLE (from Yeast) High Recovery (Takara Bio, Shiga, Japan). The concentration, integrity and purity of total extracted DNA were confirmed by gel electrophoresis in 0.8% agarose in 0.5× Tris-Borate-EDTA (TBE). The nuclear rRNA genes for SSU, ITS and D1/D2 LSU were amplified and sequenced as described previously [26]–[28]. Two protein-coding genes, *RPB1* and *XYL1*, were amplified using the following degenerate primer pairs: RPB1-Af (5′-GARTGYCCDGGDCAYTTYGG-3′) and RPB1-Cr (5′-CCNGFCDATNTCRTTRTCCATRTA-3′) for *RPB1* [29], [30]; XYL1-forward (5′-GGTYTTYGGMTGYTGGAARSTC-3′) and XYL1-reverse (5′-AAWGATTGWGGWCCRAAWGAWGA-3′) for *XYL1* [3], [5]. The PCR conditions recommended in the references for each primer pair were employed. The purified PCR products were sequenced using a Dye Terminator cycle sequencing kit (Applied Biosystems, Warrington).

### Phylogenetic Analyses

Comparisons with sequences from the international GenBank database (http://www.ncbi.nlm.nih.gov/) were done using BLASTN search. Sequences were aligned using the multiple sequence alignment program CLUSTAL X 1.83 [31]. Phylogenetic trees were constructed using the neighbour-joining and maximum parsimony programs in MEGA software version 5.0 [32]. The evolutionary distance data was calculated from Kimura’s two-parameter model [33] in the neighbour-joining analyses [34]. The heuristic search (close-neighbour-interchange) was used in the maximum parsimony analyses. The sites containing gaps in the alignments of a single gene or combined sequences were excluded. Bootstrap analyses [35] were performed from 1000 random resamplings.

### Nomenclature

The electronic version of this article in Portable Document Format (PDF) in a work with an ISSN or ISBN will represent a published work according to the International Code of Nomenclature for algae, fungi, and plants, and hence the new names contained in the electronic publication of a PLOS ONE article are effectively published under that Code from the electronic edition alone, so there is no longer any need to provide printed copies.

In addition, new names contained in this work have been submitted to MycoBank from where they will be made available to the Global Names Index. The unique MycoBank number can be resolved and the associated information viewed through any standard web browser by appending the MycoBank number contained in this publication to the prefix http://www.mycobank.org/MB. The online version of this work is archived and available from the following digital repositories: PubMed Central; LOCKSS.

## Results and Discussion

### Yeast Isolation and Diversity

A total of 105 yeast strains were isolated from rotten wood samples obtained from Baotianman Nature Reserve, central China. Initial biochemical characterization of fermentation abilities was performed on all the isolates, which showed that only 17 yeast strains had the ability to ferment D-xylose. Based on the rapid identification of the D1/D2 domain of the LSU rRNA gene, the majority of these D-xylose-fermenting yeast strains were identified as known species that included *S*. *insectosa* (2 isolates), *S*. *lignosus* (1 isolate), *S*. *segobiensis* (2 isolates), *S*. *stipitis* (3 isolates), *S*. *shehatae* (5 isolates) and *Spathaspora passalidarum* (2 isolates). The other two strains, BY-41^T^ and BY-58, were closely related to *S*. *segobiensis*, *S*. *stipitis* and other species in the *Scheffersomyces* clade and were almost indistinguishable phylogenetically from one another.

### Proposal of New Yeast Species

**Figure 1:**
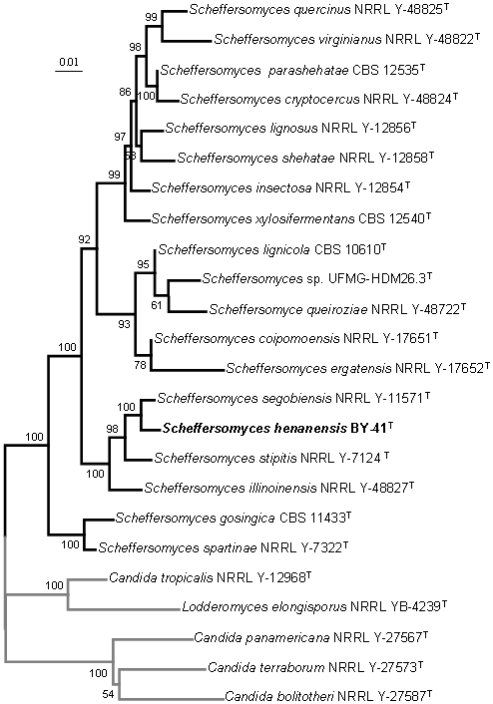
Phylogenetic tree constructed from neighbour-joining analysis of the combined sequences of SSU, ITS, D1/D2 LUS and *RPB1*, depicting the relationships of *Scheffersomyces henanensis* sp. nov. with closely related taxa in the *Scheffersomyces* clade. *Candida tropicalis* was used as an outgroup taxon (in gray). Bootstrap percentages over 50% from 1000 bootstrap replicates are shown. Bar, 0.01 substitutions per nucleotide position.

Two strains BY-41^T^ and BY-58 were found to share identical nuclear rRNA genes (SSU, ITS and LSU) and *RPB1*, indicating their conspecificity. In order to obtain a clearer phylogenetic circumscription of the novel strains and their closely related species in the *Scheffersomyces* clade, we used a multilocus phylogenetic analysis that included the traditional rRNA genes (SSU, ITS and LSU) and the orthologous *RPB1* as defined by Urbina and Blackwell [3], [5]. A phylogenetic tree constructed by the neighbour-joining method based on the multilocus sequence analysis showed that our isolates connected to *S*. *segobiensis* with 100% bootstrap support and constituted a cluster with *S*. *stipitis* and *S*. *illinoinensis* in the *Scheffersomyces* clade (Table 1 and Fig. 1). The same tree topology was derived from the maximum parsimony analysis (results not shown). The nucleotide differences between the new strains and their closest relatives, *S*. *segobiensis*, *S*. *stipitis* and *S*. *illinoinensis* are given in Table 2. These results clearly indicated that the new strains were representatives of a novel species closely related to *S*. *segobiensis*.

**Table 1:** GenBank accession numbers of the nucleotide sequences used in this study*.

Species	Codes	SSU	ITS	LSU	*RPB1*	*XYL1*
*C*. *bolitotheri*	NRRL Y-27587^T^	AY242142	FJ623599	AY242249	JN804828	–
*C*. *terraborum*	NRRL Y-27573^T^	AY426956	FJ623596	AY309810	JN804831	–
*C*. *panamericana*	NRRL Y-27567^T^	AY242164	FJ623601	AY242273	JN804835	–
*S*. *coipomoensis*	NRRL Y-17651^T^	HQ651931	HQ652070	HQ651966	KC507420	–
*S*. *lignicola*	CBS 10610^ T^	AY845351	HQ652074	AY845350	–	–
*S*. *ergatensis*	NRRL Y-17652^T^	AB013524	EU343826	U45746	EU344098	JQ436926
*S*. *insectosa*	NRRL Y-12854^T^	AB013583	HQ652064	U45773	JN804842	JQ235697
*S*. *lignosus*	NRRL Y-12856^T^	HQ651941	JN943262	U45772	JN804837	JQ235693
*S*. *segobiensis*	NRRL Y-11571^T^	AB054288	DQ409166	U45742	EF599429	JQ436925
*L*. *elongisporus*	NRRL YB-4239^T^	HQ876033	HQ876042	HQ876050	AY653537	–
*C*. *tropicalis*	NRRL Y-12968^T^	EU348785	AB437068	U45749	–	–
*S*. *queiroziae*	NRRL Y-48722^T^	–	HM566445	HM566445	–	–
*S*. *gosingicus*	CBS 11433^ T^	HQ876040	HQ999978	HQ999955	–	–
*S*. *spartinae*	NRRL Y-7322^T^	FJ153139	HQ876044	U45764	–	–
*S*. *stipitis*	NRRL Y-7124^T^	AB054280	JN943257	U45741	JN804841	JQ235696
*Scheffersomyces* sp.	NRRL Y-48762^T^	–	JF826438	JF826438		
*S*. *shehatae*	NRRL Y-12858^T^	AB013582	JN943264	JQ025409	JQ436927	JQ235691
*S*. *quercinus*	NRRL Y-48825^T^	JN940981	JN943260	JN703957	JN804838	JQ008829
*S*. *virginianus*	NRRL Y-48822^T^	JN940969	JN943259	JN703958	JN804839	JQ235695
*S*. *illinoinensis*	NRRL Y-48827^T^	JN940968	JN943261	JN703959	JN804840	JQ235694
*S*. *cryptocercus*	NRRL Y-48824^T^	JQ714001	JQ713977	JQ714021	JQ713989	JQ714031
*S. parashehatae.*	CBS 12535^T^	HQ651936	HQ652051	HQ651972	JQ023138	KC479716
*S. xylosifermentans*	CBS 12540^T^	HQ876038	HQ652061	HQ652020	JQ023142	KC479722
*S*. *henanensis*	CBS 12475^ T^	JF896577	HQ127627	HQ127626	KF690371	KF690374

*Sequences generated in this work shown in bold. ^T^  =  type strain.

**Table 2:** Nucleotide differences and percentages of homology between *Scheffersomyces henanensis* sp. nov. and the type cultures of closest relatives, S. *segobiensis*, *S*. *stipitis* and *S. illinoinensis*.

Species	SSU	ITS	D1/D2 LUS	*RPB1*	*XLY1*
*S*. *segobiensis* ^T^	99% (9 n)	99% (1 n)	99% (4 n)	96% (23 n)	96% (20 n)
*S*. *stipitis* ^T^	99% (6 n)	99% (2 n)	99% (3 n)	91% (58 n)	96% (20 n)
*S*. *illinoinensis* ^T^	99% (5 n)	99% (5 n)	98% (11 n)	91% (58 n)	95% (29 n)

**Figure 2:**
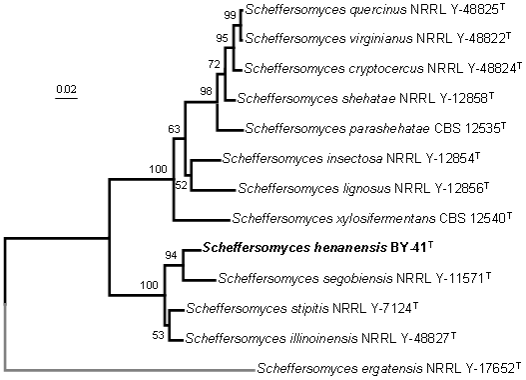
Phylogenetic tree reconstructed from neighbour-joining analysis of *XYL1* sequences depicting the relationships of *Scheffersomyces henanensis* sp. nov. with closely related taxa in *S*. *stipitis* subclade. *Scheffersomyces ergatensis* was used as an outgroup taxon (in grey). Numbers above each branch refer to bootstrap values out of 1000 repetitions. Bar, 0.02 substitutions per nucleotide position.

The sequence analysis of the easily amplified *XYL1* was recently recommended for rapid identification of cryptic species in the *Scheffersomyces* clade [3], [5]. Therefore, *XYL1* was amplified from the two strains of the proposed new species and sequenced. The *XYL1* sequences of these strains were identical with each other, but differed significantly from those of *S*. *segobiensis*, their nearest phylogenetic neighbour, by 3.8% sequence divergence (25 substitutions, 0 gaps) in 525 nt (Table 2 and Fig. 2). Phylogenetic analysis based on the nucleotide sequence of *XYL1* alone supported the separation of these strains as a unique species, as also determined by the multilocus phylogenetic construction (Table 1, Fig. 1 and 2). These results described above further confirm our provisional characterization of these strains as a new species of the genus *Scheffersomyces*.

**Figure 3:**
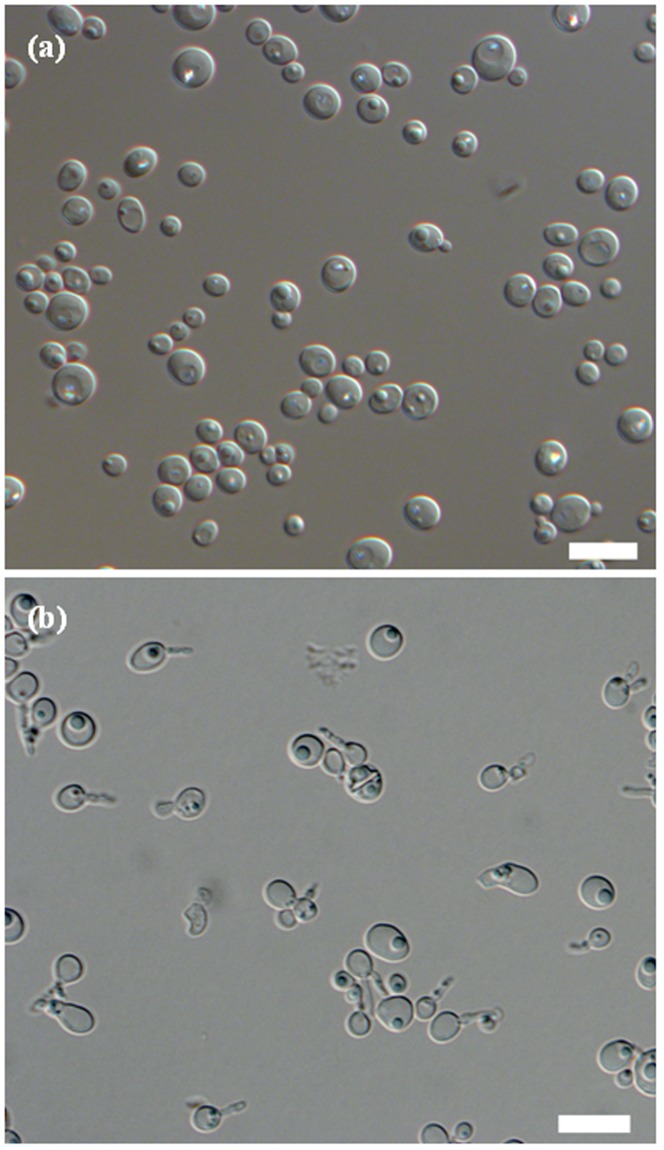
Morphological characterization of *Scheffersomyces henanensis* sp. nov. BY-41^T^. (a) Budding cells grown on YM broth for 3 days at 25°C. (b) Asci formed on cornmeal agar after 6 days at 25°C. Bar, 10 μm.

Cells of two isolates were spherical to ellipsoidal (Fig. 3a), reproduced by multilateral budding, formed one to two hat-shaped ascospores (Fig. 3b), produced pseudohyphae but not true hyphae, fermented D-xylose, gave negative diazonium blue B reaction and contained Q-9 as the major ubiquinone. These characteristics fit well with those of species of the genus *Scheffersomyces*. However, these two strains also exhibited a number of distinct physiological characteristics that clearly differentiated them from *S*. *segobiensis* and other closely related species of the genus *Scheffersomyces* (Table 3). For instance, they ferment melezitose, whereas both *S*. *stipitis* and *S*. *segobiensis* ferment trehalose. In addition, the novel strains are able to assimilate inulin, galactitol and D-galacturonic acid unlike the other *Scheffersomyces* species described to date.

**Table 3:** Physiological characteristics that differentiate *Scheffersomyces henanensis* sp. nov. from related species*.

Characteristic	*S*. *henanensis*	*S*. *stipitis*	*S*. *segobiensis*
Fermentation			
Maltose	+	+, D	–
Trehalose	–	+, D	D
Cellobiose	–	D, –	–
Melezitose	D, W	–	–
Starch	D, W	D, –	–
Assimilation			
L-Sorbose	–	D, –	D
D-Ribose	–	+, D	+
Melezitose	D	+, D	–
Inulin	+	–	–
Soluble starch	+	+	–
Erythritol	D, W	+	–
Galactitol	+	–	–
D-Gluconate	+	D, –	–
D-Galacturonic acid	+	–	–

*Data for reference species were taken from Barnett *et al*. (2000). Symbols: +, Positive; –, negative; D, delayed positive; and W, weakly positive.

On the basis of the multilocus sequence analyses of the nuclear rRNA genes and two protein-coding genes, as well as other taxonomic characteristics reported above, we conclude that the two strains represent a single novel species belonging to the genus *Scheffersomyces*. The novel species is described as *Scheffersomyces henanensis* sp. nov., with type strain BY-41^T^ ( =  CICC 1974^T^  =  CBS 12475^T^).

### Description of *Scheffersomyces henanensis* Hui, Ren, Chen & Niu sp. nov

Hui et al. 2014, sp. nov. [urn:lsid:imycobank.org:names:MB 805938.

In YM broth after 3 days at 25°C, cells are spherical or ovoid (2–6.5 ×2–7 μm) and occur singly or in pairs (Fig. 3a). Budding is multilateral. On YM agar after 3 days at 25°C, the streak culture is butyrous, white, raised with a smooth surface and has an entire margin. In Dalmau plates after 7 days on cornmeal agar at 25°C, pseudohyphae are formed, but true hyphae are not formed. On cornmeal agar and 5% malt extract agar after 6 days at 25°C, conjugated asci are formed and each ascus contains one to two hat-shaped ascospores. Asci are deliquescent (Fig. 3b). The major ubiquinone is Q-9. A summary of the physiological and other growth characteristics of *S*. *henanensis* is given in Table 4.

**Table 4:** Physiological characteristics of *Scheffersomyces henanensis* sp. nov.*

Fermentation			
D-Glucose	**+**	Inulin	–
D-Galactose	**+**	Cellobiose	–
Sucrose	–	Methyl-a-D-glucoside	–
Maltose	**+**	Melibiose	–
Lactose	–	Melizitose	D, W
Raffinose	–	Starch	D, W
α,α-Trehalose	–	D-Xylose	D
Carbon assimilation			
D-Glucose	**+**	Raffinose	–
D-Galactose	**+**	Melezitose	D
L-Sorbose	–	Inulin	**+**
D-Glucosamine	–	Soluble starch	**+**
D-Ribose	–	Glycerol	**+**
D-Xylose	**+**	Erythritol	D, W
L-Arabinose	–	Ribitol	**+**
D-Arabinose	**+**	Xylitol	**+**
L-Rhamnose	**+**	L-Arabinitol	–
Sucrose	**+**	D-Glucitol	**+**
Maltose	**+**	D-Mannitol	**+**
Trehalose	**+**	Galactitol	**+**
Methyl-a-D-glucoside	D	*myo*-Inositol	–
Cellobiose	**+**	DL-Lactate	–
Salicin	**+**	Succinate	**+**
Arbutin	**+**	Citrate	**+**
Melibiose	–	Methanol	–
Lactose	–	Ethanol	W
D-Gluconate	**+**	D-Galacturonic acid	**+**
Nitrogen assimilation			
Nitrate	–	Creatine	–
Nitrite	–	Creatinine	–
Ethylamine	**+**	Glucosamine	–
L-Lysine	**+**	Imidazole	–
Cadaverine	**+**	D-Tryptophan	–
Growth tests			
10%NaCl/5% glucose	–	0.1% Cycloheximide	**+**
50% Glucose	–	Vitamin-free medium	–
Starch formation	–	Growth at 35°C	**+**
1% Acetic acid	–	Growth at 37°C	–
Additional tests			
Starch formation	–	Urea hydrolysis	–
Acetic acid production	–	Diazonium blue B reaction	–

*Symbols: +, Positive; –, negative; D, delayed positive; and W, weakly positive.

#### Type strain

CICC 1974^T^ ( =  CBS 12475; BY-41) is preserved as a lyophilized preparation in China Center of Industrial Culture Collection (CICC), Beijing, China, and the Yeast Collection of the Centraalbureau voor Schimmelcultures (CBS), Utrecht, the Netherlands. The strain was isolated from rotten wood collected in August 2009 from Baotianman Nature Reserve in Henan Province, central China, the coordinates for which are 33°27′47′′N and 111°48′32′′E.

#### Etymology

The species name *henanensis* (he.nan.en'sis. L. nom. masc. adj.) refers to Henan Province, central China, the geographical origin of the species.
